# Supplementary material for: Local and Systemic Adverse Reactions to mRNA COVID-19 Vaccines Comparing Two Vaccine Types and Occurrence of Previous COVID-19 Infection
Source: Vaccines (Basel). 2021 Dec 10;9(12):1463. doi: 10.3390/vaccines9121463 (PMC8707814; doi:10.3390/vaccines9121463)
Supplement: Supplementary file 1 [file vaccines-09-01463-s001.zip › vaccines-1477020-supplementary.pdf]

Supplementary Materials

Annex S1. Questionnaire

- Date of birth
- Sex: Women, Men, Non-binary
- Worker category: medical doctor, registered nurse, other (with patient contact), other (without patient contact).
- Have you ever experienced a severe allergy (anaphylactic shock or glottis edema)?: yes, no
- What caused this reaction?: medications, other
- Do you have or have had a chronic condition (such as cardiac insufficiency, ischemic heart disease, asthma, diabetes, chronic bronchitis, neurological disease, renal failure or chronic liver disease)?: yes, no
- Have you ever had COVID-19?: yes, no
- Date of COVID-19 diagnosis
- How serious were your COVID-19 symptoms?: asymptomatic infection, mild or moderate symptoms, hospitalization
- Which COVID-19 vaccine did you receive: Pfizer, Moderna, I don’t know
- Date of vaccination
- Did you have adverse reactions to COVID-19 vaccine first dose: yes, no
- Which adverse reactions did you suffer (multiple choice):
    - Pain at injection site
    - Swelling or redness at injection site
    - Fatigue
    - Headache
    - Muscle or join pain
    - Chills
    - Fever (>=37.5º)
    - Nausea or vomiting
    - Adenopathy / swollen lymph nodes
    - Insomnia
    - Malaise
    - Hives or rash
- Date of onset of adverse reaction
- Date of end of adverse reaction
- Did you need a medical leave because of the adverse reactions?: yes, no
- Did you need medical attention because of the adverse reactions?: yes, no
- Was the adverse reaction life-threatening?: yes, no
- Do you allow us to access your clinical history?: yes, no
    - What is your health record ID code?

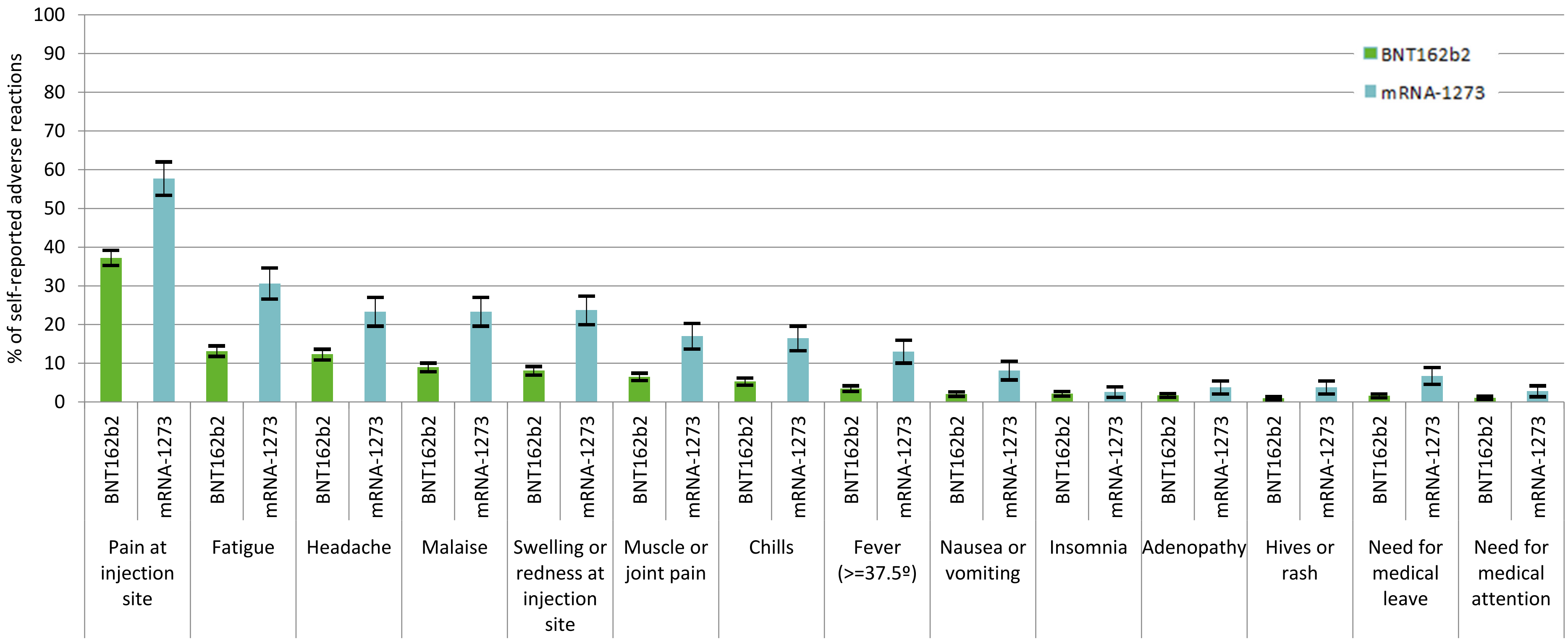

Annex S2: Percentage of self-reported adverse reactions by vaccine type, dose 1

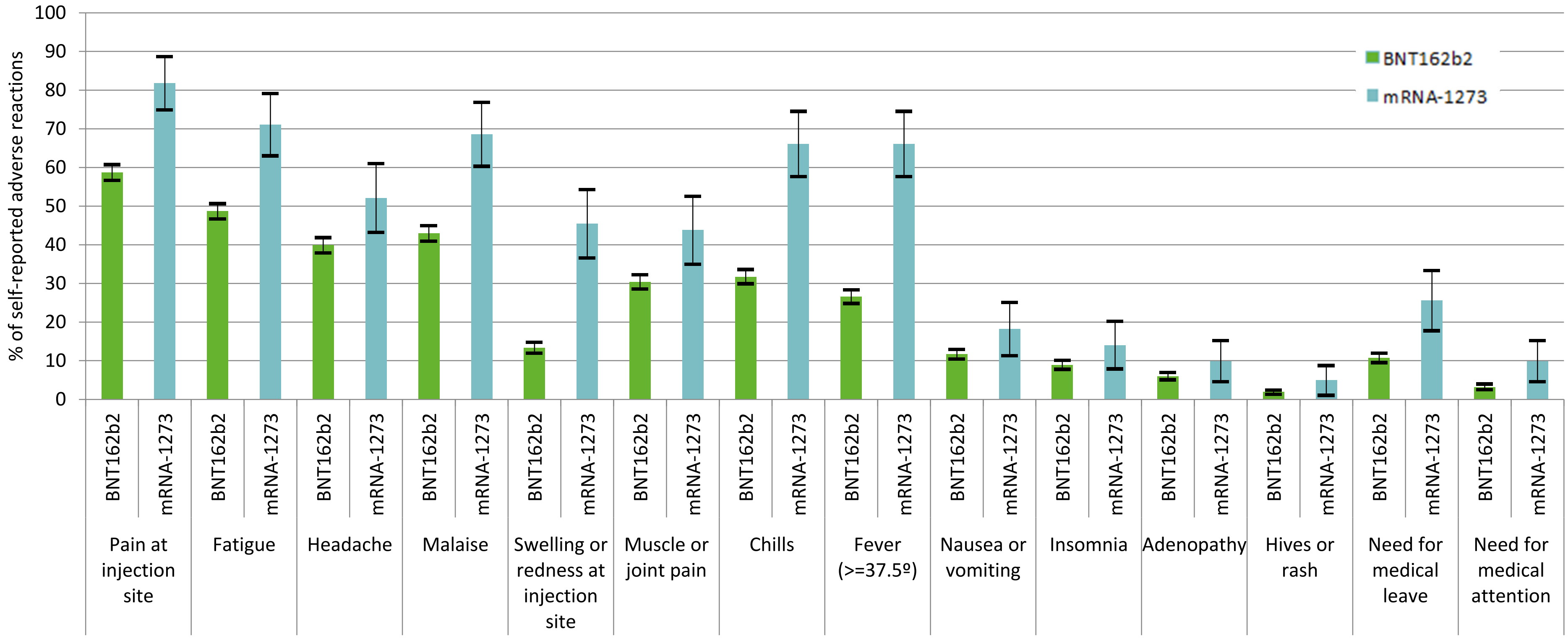

Annex S3: Percentage of self-reported adverse reactions by vaccine type, dose 2
